# Crystal structures of main proteases of SARS-CoV-2 variants bound to a benzothiazole-based inhibitor: Crystal structures of M ^pro^ mutants bound to YH-53

**DOI:** 10.3724/abbs.2023053

**Published:** 2023-06-26

**Authors:** Jiqing Luo, Weiwei Wang, Haihai Jiang, Wenwen Li, Pei Zeng, Jie Wang, Xuelan Zhou, Xiaofang Zou, Shenghui Chen, Qisheng Wang, Jin Zhang, Jian Li

**Affiliations:** 1 College of Pharmaceutical Sciences Gannan Medical University Ganzhou 341000 China; 2 Shanghai Advanced Research Institute Chinese Academy of Sciences Shanghai 201204 China; 3 School of Basic Medical Sciences Nanchang University Nanchang 330031 China; 4 Shenzhen Crystalo Biopharmaceutical Co. Ltd. Shenzhen 518118 China; 5 Jiangxi Jmerry Biopharmaceutical Co. Ltd. Ganzhou 341000 China; 6 Nanchang Reproductive Hospital Nanchang 330000 China

**Keywords:** SARS-CoV-2, variant, main protease, inhibitor, benzothiazole, YH-53

## Abstract

Main protease (M ^pro^) serves as an indispensable factor in the life cycle of severe acute respiratory syndrome coronavirus 2 (SARS-CoV-2) as well as its constantly emerging variants and is therefore considered an attractive target for antiviral drug development. Benzothiazole-based inhibitors targeting M ^pro^ have recently been investigated by several groups and proven to be promising leads for coronaviral drug development. In the present study, we determine the crystal structures of a benzothiazole-based inhibitor, YH-53, bound to M ^pro^ mutants from SARS-CoV-2 variants of concern (VOCs) or variants of interest (VOIs), including K90R (Beta, B.1.351), G15S (Lambda, C.37), Y54C (Delta, AY.4), M49I (Omicron, BA.5) and P132H (Omicron, B.1.1.529). The structures show that the benzothiazole group in YH-53 forms a C-S covalent bond with the sulfur atom of catalytic residue Cys145 in SARS-CoV-2 M ^pro^ mutants. Structural analysis reveals the key molecular determinants necessary for interaction and illustrates the binding mode of YH-53 to these mutant M ^pro^s. In conclusion, structural insights from this study offer more information to develop benzothiazole-based drugs that are broader spectrum, more effective and safer.

## Introduction

The outbreak of coronavirus disease-19 (COVID-19) in 2019 turned into a global pandemic and seriously impacted the public health system and economy [ 1, 2] . Severe acute respiratory syndrome coronavirus-2 (SARS-CoV-2) was identified as the causative agent soon after the COVID-19 outbreak [3]. This agent belongs to the β-coronavirus genus, which also includes SARS-CoV and MERS-CoV [ 4, 5] . Before the SARS-CoV-2 outbreak, SARS-CoV and MERS-CoV caused the most recent two other coronavirus outbreaks that occurred in China in 2003 [6] and in Saudi Arabia in 2012 [7], respectively. Unlike the extinguished outbreak of SARS-CoV and MERS-CoV, the SARS-CoV-2 outbreak has lasted for three years and has caused 753 million cases of COVID-19 as of January 31, 2023, including 6.8 million deaths ( https://covid19.who.int/).

The biomedical research community has rapidly started the study on vaccines since the SARS-CoV-2 outbreak. Several vaccines developed by various technical methods have been approved by different countries and put into market [ 8‒ 10] , playing a positive role in combating the COVID-19 pandemic. However, SARS-CoV-2 is easy to mutate, and the emerged variants cause concern about virus characteristics, including transmissibility and antigenicity. Five variants have been identified by the WHO as Variants of concern (VOC), namely, B.1.1.7 (Alpha, α), B.1.351 (Beta, β), P.1 (Gamma, γ), B.1.617.2 (Delta, δ), and B.1.1.529 (Omicron), and two variants have been identified as Variants of Interest (VOI), including C.37 (Lambda, λ) and B.1.621 (Mu, μ) ( https://www.who.int/activities/tracking-SARS-CoV-2-variants). Because of the existence of persistent mutations, the effectiveness of current vaccines may be compromised. In addition to vaccines, mankind urgently needs broad-spectrum drugs to treat COVID-19.

To develop an antiviral drug against SARS-CoV-2, a series of studies evaluated the potential drug targets of SARS-CoV-2. Among them, the main protease (M ^pro^) is one of the foremost appealing targets in the life cycle and pathogenicity of the virus [ 11‒ 13] . SARS-CoV-2 M ^pro^ is an indispensable enzyme in viral replication and transcription [14], and it is extremely critical for the cleavage of two polypeptide (pp1a and pp1ab) sequences after a glutamine residue [13]. It has a unique feature of substrate recognition that is not shared by human proteases [ 13, 15, 16] . Moreover, M ^pro^ is highly conserved in SARS-CoV-2, which also makes it possible to develop broad-spectrum coronavirus drugs [ 17‒ 19] . In summary, M ^pro^ is an important and conserved target for the inhibition of SARS-CoV-2. Inhibitors targeting M ^pro^ can effectively prevent the replication of SARS-CoV-2 and therefore represent promising drug candidates.

Over a sustained period, particularly from the start of the pandemic, drug repurposing has been a successful alternative to drug development [ 20, 21] . Therefore, previously effective small molecule inhibitors against M ^pro^ are potential pools to combat the coronavirus disease COVID-19 [ 22‒ 25] . Among many researched inhibitors, the benzothiazole-based small molecule YH-53 shows exciting prospects [26]. YH-53 is a peptidomimetic inhibitor that effectively inhibits the action of M ^pro^ in SARS-CoV-2 in enzymatic and cellular antiviral assays [ 26‒ 29] . YH-53 offers a number of advantages, including good metabolic and excretion profile, good pharmacokinetics and no apparent toxicity [ 26, 28] . As a covalent inhibitor, YH-53 primarily utilizes the benzothiazole moiety as a warhead, and it is derived from a tetrapeptide inhibitor by the addition of a trifluoromethyl ketone fraction [26]. The benzothiazole group (electron attracting group) can be greatly enhanced to form covalent bonds with the M ^pro^ active site, temporarily inactivating the enzyme and completely blocking the replication of SARS-CoV-2 [ 26, 29] . These studies revealed that YH-53 can establish a covalent bond with the active site of M ^pro^, which suggests that it has the potential as a lead candidate for further drug development [26]. Our previous studies mainly focused on the enzymatic inhibition of YH-53 against M ^pro^s from different coronaviruses, including SARS-CoV, MERS-CoV and SARS-CoV-2, and illustrated the structural basis for the inhibition [29].

In the present study, we investigated the details of the interaction between the benzothiazole-based small molecule YH-53 and the mutant M ^pro^s, each carrying a single mutation, by solving the complex structures. These mutations come from different SARS-CoV-2 variants: B.1.351 Beta (K90R), C.37 Lambda (G15S), Delta AY.4 (Y54C), BA.5 Omicron (M49I) and B.1.1.529 Omicron (P132H). The results provided structural insights for understanding the precise inhibition mechanism of the inhibitor against different M ^pro^s and strongly indicated the potential of YH-53 to be developed as a broad-spectrum drug candidate. Additionally, the present study will ultimately provide theoretical guidance for finding alternatives to treat different variants of SARS-CoV-2.

## Materials and Methods

### Expression and purification of M ^pro^ mutants from human SARS-CoV-2

A plasmid containing the gene encoding wild-type SARS-CoV-2 M ^pro^ was constructed based on a previous method [29]. Using the wild-type plasmid as a template, site-directed mutagenesis was performed to form each of the variations (K90R, G15S, Y54C, M49I and P132H). Nucleotide sequences were confirmed by DNA sequencing. The plasmids containing genes encoding mutant M ^pro^s were introduced into competent cells *E*. *coli* Rosetta DE3 for protein expression. The expression and purification of the M ^pro^ mutants with His-tag were performed according to the method described previously [18]. Furthermore, TEV protease was used to remove the N-terminal His-tag.

### Crystallization of various M ^pro^ mutants in complex with YH-53

Five mutants of SARS-CoV-2 M ^pro^ were concentrated to 5 mg/mL, and incubated on ice with YH-53 at a molar ratio of 1:3 for 30 min. Crystallization was performed at 18°C using the hanging drop vapour-diffusion method. After 3 to 5 days, crystals of YH-53 in complex with five mutant M ^pro^s were obtained. The final crystallization condition of the SARS-CoV-2 M ^pro^ (K90R)-YH-53 complex was 0.1 M HEPES, pH 7.5, 18% (w/v) PEG 10000; the final crystallization condition of the SARS-CoV-2 M ^pro^ (G15S)-YH-53 complex was 0.1 M HEPES, pH 7.5, 18% (w/v) PEG 10000; the final crystallization condition of the SARS-CoV-2 M ^pro^ (Y54C)-YH-53 complex was 0.1 M HEPES pH 7.5, 18% (w/v) PEG 10000; the final crystallization condition of the SARS-CoV-2 M ^pro^ (M49I)-YH-53 complex was 0.1 M HEPES, pH 7.5, 18% (w/v) PEG 10000; and the final crystallization condition of the SARS-CoV-2 M ^pro^ (P132H)-YH-53 complex was 0.1 M HEPES, pH 7.5, 18% (w/v) PEG 10000.

### Data collection, structure determination, and refinement

The crystals were stabilized and cryoprotected by the addition of a reservoir solution containing 20% glycerol and then flash cooled in liquid nitrogen. All X-ray diffraction data sets were collected at 100 K at BL10U2 of the Shanghai Synchrotron Radiation Facility (Shanghai, China). Diffraction data were autoprocessed using the aquarium pipeline 24 [30], and the data processing statistics are listed in Table 1. All the structures of M ^pro^ mutants in complex with YH-53 were determined by the molecular replacement method using the program Phaser [31]. The maximum likelihood-based refinement of the atomic positions and temperature factors was performed with Phenix [32]. The atomic model was fit with the program Coot [33]. The stereochemical quality of the final model was assessed with MolProbity [34]. The structural refinement statistics of the mutant M ^pro^s from SARS-CoV-2 variants in complex with YH-53 are shown in Table 1. Figures were prepared with PyMOL.

**Table:** **Table 1** Data collection and refinement statistics

Item	SARS-Cov-2 K90R -YH53	SARS-Cov-2-G15S-YH53	SARS-Cov-2 Y54C-YH53	SARS-Cov-2 M49I-YH53	SARS-Cov-2 P132H-YH53
Data collection	8HQG	8HQF	8HQJ	8HQH	8HQI
Space group	*P1211*	*P1211*	*P1211*	*P1211*	*P1211*
a, b, c (Å)	55.13, 99.00, 59.32	55.38, 99.44, 59.46	55.60, 99.46, 59.80	55.42, 99.14, 59.67	55.58, 99.45, 59.37
α, β, γ (°)	90.00, 108.05, 90.00	90.00, 108.25, 90.00	90.00, 108.13, 90.00	90.00, 108.57, 90.00	90.00, 108.30, 90.00
Wavelength (Å)	0.97918	0.97918	0.97918	0.97918	0.97918
Resolution (Å) ^a^	1.88 (1.98‒1.88)	1.51 (1.59‒1.51)	1.74 (1.84‒1.74)	1.58 (1.66‒1.58)	1.55 (1.64‒1.55)
Total reflections	310954	582294	345265	531476	486986
Unique reflections ^a^	45864	93541	58120	84233	81204
*R*merge (%) ^a^	3.4 (67.4)	2.9 (63.5)	2.9 (70.2)	2.9 (60.4)	5.5 (79.7)
Mean I/σ (I) ^a^	14.3 (2.5)	19.6 (2.5)	16.6 (2.7)	18.8 (2.5)	13.2 (2.5)
Completeness (%) ^a^	93.0 (93.1)	98.0 (96.6)	92.4 (100.0)	100.0 (100.0)	92.2 (100.0)
Multiplicity ^(a)^	6.8 (6.8)	6.2 (4.8)	5.9 (5.8)	6.3 (5.1)	6.0 (4.8)
Refinement					
Resolution (Å)	49.10‒1.88	49.10‒1.51	52.84‒1.74	49.57‒1.58	52.77‒1.55
*R*_work_/ *R*_free_^b^	21.70/26.20	20.84/23.07	20.52/23.52	21.23/22.75	21.72/23.92
Atoms	4616	4709	4716	4676	4711
Mean temperature factor (Å ^2^)	33.9	23.1	31.0	26.7	24.8
Bond lengths (Å)	0.007	0.006	0.006	0.006	0.006
Bond angles (°)	0.954	0.941	0.904	0.973	0.887
Ramachandran plot					
Favored (%)	97.64	98.31	98.28	98.31	98.14
Allowed (%)	2.36	1.69	1.72	1.69	1.86
Outliers (%)	0	0	0	0	0

**^a^** The values in parentheses are for the outermost shell.

**^b^** R _free_ is the *R*_work_ based on 5% of the data excluded from the refinement.

### Data availability

Structure factors and coordinates have been deposited in the Protein Data Bank under accession codes 8HQG for SARS-CoV-2 M ^pro^ (K90R)-YH-53, 8HQF for SARS-CoV-2 M ^pro^ (G15S)-YH-53, 8HQJ for SARS-CoV-2 M ^pro^ (Y54C)-YH-53, 8HQH for SARS CoV-2-M ^pro^ (M49I)-YH-53 and 8HQI for SARS-CoV-2 M ^pro^ (P132H)-YH-53.

## Results

### Expression and purification of M ^pro^ mutants

**Figure 1:**
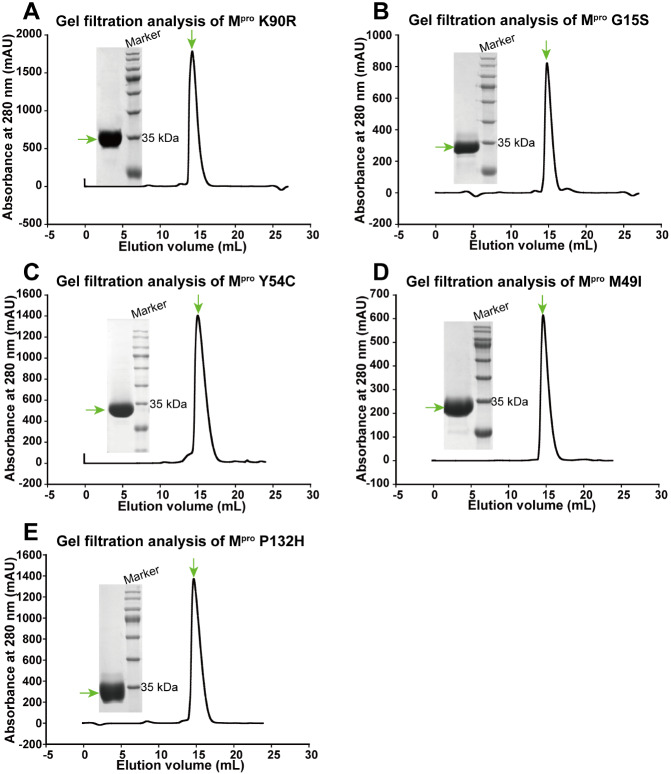
Figure 1 Purification of SARS-CoV-2 M ^pro^ mutants Gel filtration was used to purify the SARS-CoV-2 M pro K90R (A), G15S (B), Y54C (C), M49I (D), and P132H (E). A Superdex 200 Increase 10/300 GL column was used to perform gel filtration. The elution fractions were pooled and analyzed by SDS-PAGE.

All five mutant M ^pro^s were expressed in *E*. *coli* cells and purified to homogeneity by gel filtration. The elution volume and molecular weight (approximately 35 kDa) of each M ^pro^ mutant were almost identical ( Figure 1), which was also observed for wild-type M ^pro^. These data indicated that these mutant M ^pro^s share similar protein folding efficiency with the wild-type M ^pro^.

### Crystal structures of YH-53 in complex with M ^pro^ mutants from SARS-CoV-2 variants

**Figure 2:**
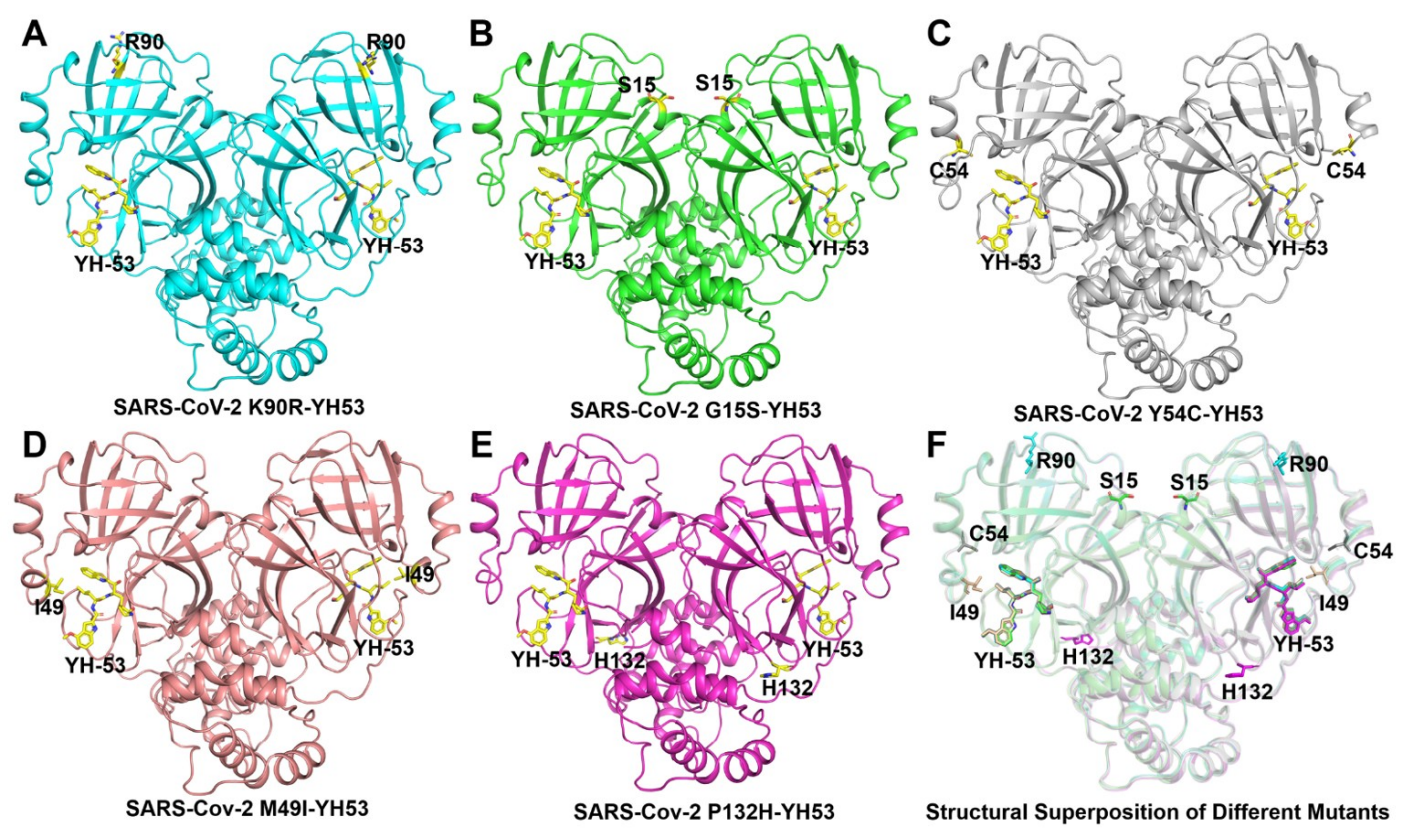
Figure 2 Structural overview of M ^pro^ mutants from SARS-CoV-2 in complex with YH-53 (A) Overall structure of the M pro K90R-YH53 complex. YH-53 is displayed as a yellow stick, while the K90R mutant is displayed as a cyan cartoon. (B) Overall structure of the M pro G15S-YH53 complex. YH-53 is displayed as a yellow stick, while the G15S mutant is displayed as a green cartoon. (C) Overall structure of the M pro Y54C-YH53 complex. YH-53 is displayed as a yellow stick, while the Y54C mutant is displayed as a gray cartoon. (D) Overall structure of the M pro M49I-YH53 complex. YH-53 is displayed as a yellow stick, while the M49I mutant is displayed as a salmon cartoon. (E) Overall structure of the M pro P132H-YH53 complex. YH-53 is displayed as a yellow stick, while the P132H mutant is displayed as a magenta cartoon. (F) Structural superposition of M pro mutant-YH-53 complexes.

We then determined the crystal structures of M ^pro^ mutants of SARS-CoV-2 in complex with YH-53 using the cocrystallization method. These structures were solved to resolutions of 1.88 Å (M ^pro^ K90R with YH-53), 1.51 Å (M ^pro^ G15S with YH-53), 1.74 Å (M ^pro^ Y54C with YH-53), 1.58 Å (M ^pro^ M49I with YH-53), and 1.51 Å (M ^pro^ P132H with YH-53). Data collection and refinement statistics are displayed in Table 1. In the five complex structures, each SARS-CoV-2 M ^pro^ mutant molecule displays a dimer form, which is also the form with enzyme activity. Overall, each protomer of the SARS-CoV-2 M ^pro^ mutant can bind to one YH-53. To compare the conformational changes of different mutants bound to YH-53, these structures were superposed. The results showed that the conformation of the five M ^pro^ mutants did not change significantly when binding with YH-53 ( Figure 2).

### Comparison of the binding modes of YH-53 with different M ^pro^ mutants

**Figure 3:**
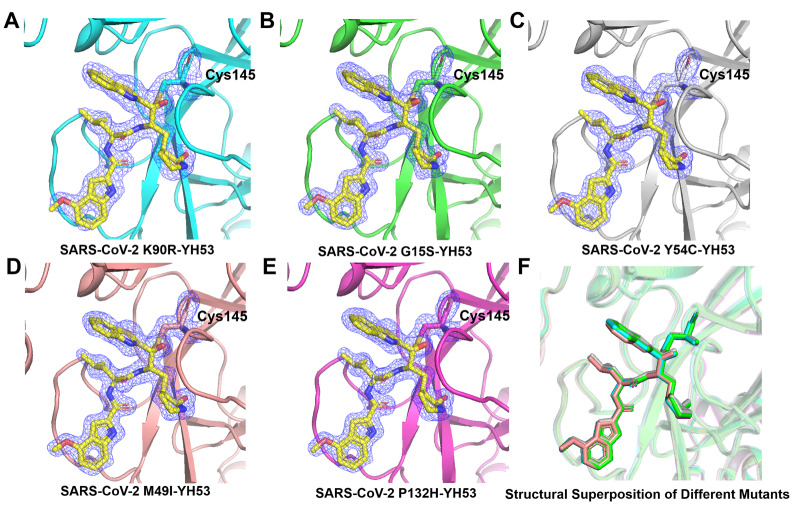
Figure 3 The 2 *Fo-Fc* electron density maps of YH-53 bound to different SARS-CoV-2 M ^pro^ mutants 2 Fo-Fc electron density maps (blue) were contoured at 1 σ. (A) The 2 Fo-Fc electron density map of YH-53 bound to the K90R mutant. (B) The 2 Fo-Fc electron density map of YH-53 bound to the G15S mutant. (C) The 2 Fo-Fc electron density map of YH-53 bound to the Y54C mutant. (D) The 2 Fo-Fc electron density map of YH-53 bound to the M49I mutant. (E) The 2 Fo-Fc electron density map of YH-53 bound to the P132H mutant. (F) An enlarged view of the structural superposition of M pro mutant-YH-53 complexes.

To better understand the binding mode of YH-53 with different mutants, we extracted the electron density map of YH-53 and the catalytic residue Cys145 ( Figure 2). The electron density diagram showed that the benzothiazole group of YH-53 is close to the sulfur atom of the catalytic residue Cys145 in the mutant SARS-CoV-2 M ^pro^, which promotes the formation of the covalent bond ( Figure 3). Cys145 is a nucleophilic reagent in the hydrolysis process of SARS-CoV-2 M ^pro^ mutants, and the binding of YH-53 inhibits their hydrolysis reaction. The inhibitor binding sites (catalytic active sites) of the five mutants were superposed to compare the structural differences caused by the M ^pro^ mutation when bound to YH-53. The result shows that there are slight differences in the orientation of YH-53 in the substrate-binding sites of different M ^pro^ mutants ( Figure 3).

### Detailed interaction between YH-53 and different M ^pro^ mutants

**Figure 4:**
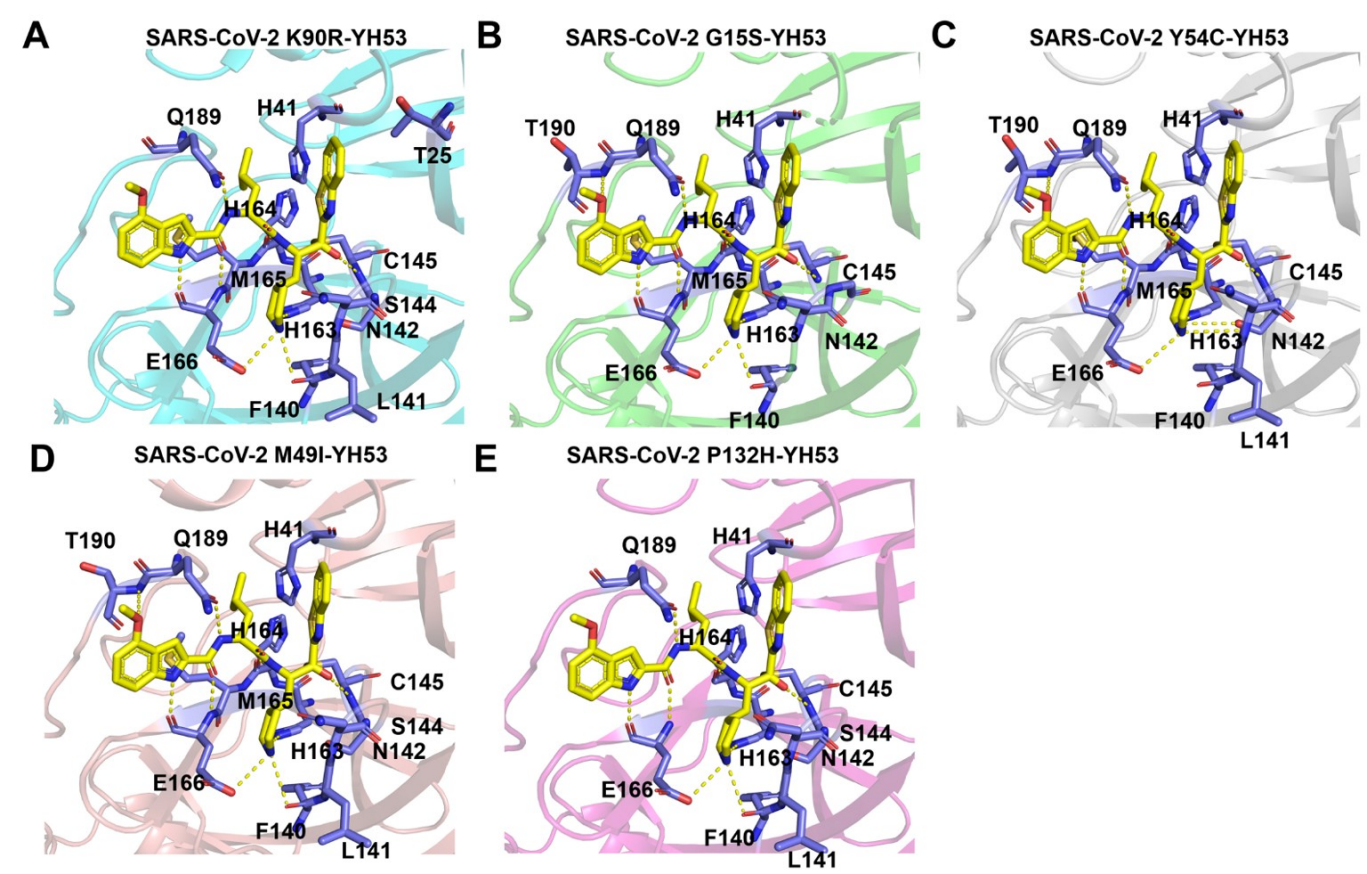
Figure 4 Interaction details between different M ^pro^ mutants and YH-53 (A) Interaction between SARS-CoV-2 M pro K90R and YH-53. His163, Glu166, Phe140, Cys145, His164 and Gln189 form hydrogen bond interactions (yellow dashed lines) with YH-53, while Thr25, His41, Leu141, Asn142, Ser144 and Met165 form hydrophobic interactions with YH53. Cys145 is also covalently bonded to the benzothiazole group of YH-53. (B) Interaction between SARS-CoV-2 M pro G15S and YH-53. Thr190, Glu166, His163, Phe140, Cys145, His164 and Gln189 form hydrogen bonds (yellow dashed lines) with YH-53. His41, Asn142 and Met165 form hydrophobic interactions with YH53. Cys145 is also covalently bonded to the benzothiazole group of YH-53. (C) Interaction between SARS-CoV-2 M pro Y54C and YH-53. Thr190, His163, Glu166, Leu141, Asn142, Cys145, His164 and Gln189 form hydrogen bonds (yellow dashed lines) with YH-53. His41, Phe140 and Met165 form hydrophobic interactions with the benzothiazole group of YH-53. Cys145 is also covalently bonded to the benzothiazole group of YH-53. (D) Interaction between SARS-CoV-2 M pro M49I and YH-53. Thr190, Glu166, His163, Phe140, Cys145, His164 and Gln189 form hydrogen bonds (yellow dashed lines) with YH-53. His41, Leu141, Asn142, Ser144 and Met165 form hydrophobic interactions with YH-53. Cys145 is also covalently bonded to the benzothiazole group of YH-53. (E) Interaction between SARS-CoV-2 M pro P132H and YH-53. Gln189, Glu166, His163, Phe140, Cys145 and His164 form hydrogen bonds (yellow dashed lines) with YH-53. His41, Leu141, Asn142 and Ser144 form hydrophobic interactions with YH53. Cys145 is covalently bonded to the benzothiazole group of YH-53.

To clearly study the interaction details between YH-53 and different mutants, the amino acids interacting with YH-53 were clearly marked with stick pattern. The interaction details between M ^pro^ K90R and YH-53 show that YH-53 interacts with several residues, including Thr25, His41, Phe140, Leu141, Asn142, Ser144, Cys145, His163, His164, Met165, Glu166, and Gln189 ( Figure 4A). The interaction details between M ^pro^ G15S and YH-53 show that YH-53 can interact with residues His41, Phe140, Asn142, Cys145, His163, His164, Met165, Glu166, Gln189, and Thr190 ( Figure 4B). The interaction details of M ^pro^ Y54C and YH-53 show that YH-53 can interact with residues His41, Phe140, Leu141, Asn142, Cys145, His163, His164, Met165, Glu166, Gln189, and Thr190 ( Figure 4C). The interaction details between M ^pro^ M49I and YH-53 show that YH-53 can interact with residues His41, Phe140, Leu141, Asn142, Ser144, Cys145, His163, His164, Met165, Glu166, Gln189, and Thr190 ( Figure 4D). The interaction details between M ^pro^ P132H and YH-53 show that YH-53 can interact with residues His41, Phe140, Leu141, Asn142, S144, Cys145, His163, His164, Glu166, Gln189, and Thr190 ( Figure 4E). Although the interaction details between different M ^pro^ mutants and YH-53 are slightly different, the catalytic dyads, Cys145 and His41, in all mutants involve the ligand-enzyme interaction.

**Figure 5:**
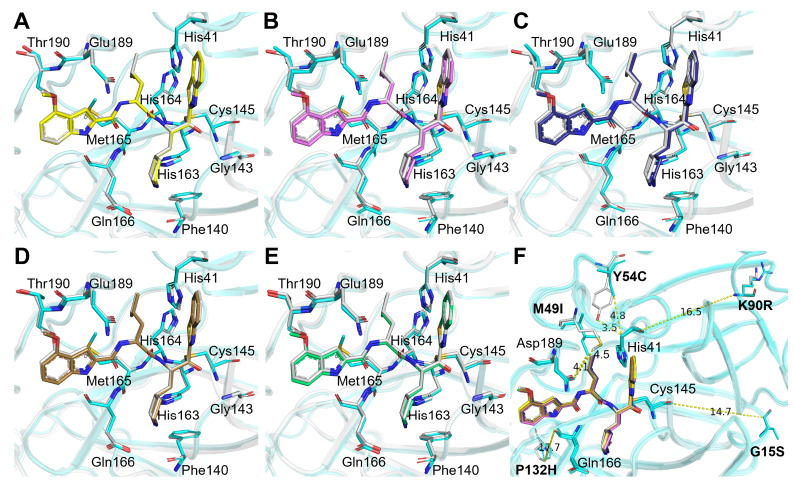
Figure 5 Structural characterization of YH-53 bound to SARS-CoV-2 M ^pro^ mutants (A–E) Superposition of the crystal structures of YH-53 bound to wild-type SARS-CoV-2 M pro (gray) and SARS-CoV-2 M pro K90R (in yellow and cyan) (A), G15S (in magenta and cyan) (B), Y54C (in deep blue and cyan) (C), M49I (in sand and cyan) (D), and P132H (in green and cyan) (E). (F) Ribbon figure of the five mutant-YH-53 crystal structures superposed to the wild-type-YH-53 complex, with the location and distance of the mutant residues (in stick representation) relative to the YH-53-binding site highlighted.

We also superimposed these mutant M ^pro^-YH-53 complex structures with wild-type M ^pro^-YH-53 complex. The results showed that there was no significant difference between wild-type and mutant M ^pro^s bound to YH-53, as shown in Figure 5A‒E. In addition, we found that mutations K90R, G15S and P132H were located at the distal end of the YH-53 binding site, while mutations Y54C and M49I were located at the proximal end of the YH-53 binding site ( Figure 5F). The crystal structures also show that these mutations will not cause significant impact on the binding pattern of YH-53 ( Figure 5F).

## Discussion

In the past three years, the COVID-19 pandemic has continued, causing serious social, economic, and political chaos. Like other RNA viruses, SARS-CoV-2 is constantly changing through mutation, and each virus with a unique sequence is considered to be a new variant. It is an urgent task for the scientific and pharmaceutical communities to develop effective drugs or therapies against SARS-CoV-2 and its variants. Among several structural and nonstructural SARS-CoV-2 proteins, M ^pro^ has been designated as a potential therapeutic target for drug development. Inhibiting M ^pro^ will prevent virus replication and constitutes one of the potential anti-coronavirus strategies. In recent years, a large number of studies have focused on the use of old drugs as a reasonable treatment for COVID-19. The success of drug reuse has attracted extensive research interest. Here, we evaluated the details of the interactions between the benzothiazole-based small molecule YH-53 and five different M ^pro^ mutants. These mutations exist in different SARS-CoV-2 variants: B.1.351 Beta (K90R), C.37 Lambda (G15S), Delta AY. 4 (Y54C), BA. 5 Omicron (M49I) and B.1.1.529 Omicron (P132H).

The crystal structures of different M ^pro^ mutants of SARS-CoV-2 bound to YH-53 were analyzed. In terms of overall structure, no significant changes in the conformation of the five M ^pro^ mutants when bound to YH-53 were observed. As indicated by the electron density map of YH-53 and the catalytic residue Cys145, there was little difference in the orientation of the benzothiazole group of YH-53. We marked the amino acids interacting with YH-53 in different mutants with the stick pattern and found that although the residues contributing to contacts with YH-53 in different M ^pro^ mutants were slightly different, the catalytic dyads (Cys145 and His41) in all mutants were involved in the interaction. As it is believed that the molecules that interact strongly with these catalytic residues are the key to establishing strong binding with and inhibition against this enzyme, the present results thus indicate that YH-53 may retain potent inhibition against these SARS-CoV-2 M ^pro^ variants.

The continuous emergence of SARS-CoV-2 variants has seriously endangered public health safety. Continuous research on inhibitors is of great benefit to quickly understand their current and future antiviral effects. Our research showed that the binding mode of YH-53 is not affected by the tested mutants, indicating that YH-53 may retain inhibitory efficacy against M ^pro^ mutants. Therefore, the present study provides a theoretical basis for the treatment of SARS-CoV-2 variants and further drug development.
